# Determinants of staff job satisfaction of caregivers in two nursing homes in Pennsylvania

**DOI:** 10.1186/1472-6963-6-60

**Published:** 2006-05-24

**Authors:** Nicholas G Castle, Howard Degenholtz, Jules Rosen

**Affiliations:** 1 Department of Health Policy and Management, University of Pittsburgh, Pittsburgh, USA; 2 Department of Psychiatry, University of Pittsburgh, Pittsburgh, USA

## Abstract

**Background:**

Job satisfaction is important for nursing home staff and nursing home management, as it is associated with absenteeism, turnover, and quality of care. However, we know little about factors associated with job satisfaction and dissatisfaction for nursing home workers.

**Methods:**

In this investigation, we use data from 251 caregivers (i.e., Registered Nurses, Licensed Practical Nurses, and Nurse Aides) to examine: job satisfaction scores of these caregivers and what characteristics of these caregivers are associated with job satisfaction. The data were collected from two nursing homes over a two and a half year period with five waves of data collection at six-month intervals. The Job Description Index was used to collect job satisfaction data.

**Results:**

We find that, overall nursing home caregivers are satisfied with the work and coworkers, but are less satisfied with promotional opportunities, superiors, and compensation. From exploratory factor analysis three domains represented the data, pay, management, and work. Nurse aides appear particularly sensitive to the work domain. Of significance, we also find that caregivers who perceived the quality of care to be high have higher job satisfaction on all three domains than those who do not.

**Conclusion:**

These results may be important in guiding caregiver retention initiatives in nursing homes. The finding for quality may be especially important, and indicates that nursing homes that improve their quality may have a positive impact on job satisfaction of staff, and thereby reduce their turnover rates.

## Background

Job satisfaction is defined as "the favorableness or unfavorableness with which employees view their work" [1]. Determinants of these views include the work environment and the personality of workers. In operationalizing the work environment, it is most often split into multiple factors (or domains) such as supervisors, pay, and promotion opportunities. In this investigation, we use a large sample of data from caregivers (i.e., nurse aides (NAs); Licensed Practical Nurses (LPNs); Registered Nurses (RNs)) working in nursing homes to examine: (1) their job satisfaction scores on 13 questions; and, (2) what characteristics of these caregivers are associated with job satisfaction domains.

### Significance of job satisfaction

Job satisfaction is clearly important for nursing home staff. Previous studies have shown a consistent association between job satisfaction and turnover and intent to turnover [e.g., [2,3]]. Given the chronic and large degree of staff turnover that currently exists in nursing homes understanding job satisfaction is important [4].

Not all dissatisfied employees leave the organization, even if they report they intend to leave. Dissatisfied employees often exhibit an unreliable work ethic, including taking unscheduled days off and tardiness. Moreover, dissatisfied employees may also show more aggression towards other workers [5] or residents [3].

Job satisfaction is also probably associated with quality of resident care [6,7]. Although not examining a long-term care setting, one study found a positive association between patient mortality and poor staff satisfaction [8]. Customer rated quality of care has a positive association with staff satisfaction [9,10]. Resident satisfaction is also associated with staff satisfaction [11]. Some recent work would also suggest that job satisfaction may be related to an organizations ability to change [12]. Since a consistent theme in the literature for the past 20 years (or more) has been the level of poor quality provided by some nursing homes [e.g., [13,14]] and their inability to change in a meaningful way, in this context improving staff satisfaction may be important in improving some aspects of quality.

Applicant attraction theories, which seek to explain how workers come to work in specific settings, would also suggest that factors promoting job satisfaction can be significant for attracting new employees to the organization [15]. As the U.S. population ages we will need more caregivers, yet an inadequate number of new caregivers are entering the healthcare workforce [16]. The General Accounting Office [4] gave one reason for this shortage as "decreased job satisfaction." This shortage of workers is clearly significant for the nursing home industry, and needs to be addressed if the industry is to attract adequate staff in the future to meet the expected demand.

### Prior research

Much of our understanding of job satisfaction comes from the business literature, and those studies set in health care facilities have predominantly examined RNs [e.g., [17,18]]. The determinants of job satisfaction may be very different for caregivers working in nursing homes. Despite the importance of job satisfaction, studies examining the determinants of job satisfaction in nursing homes are sparse. These studies and the factors of job satisfaction they examined are shown in Table 1. Parsons and associates [3], for example, found four factors associated with NA job satisfaction were: personal opportunity, supervision, benefits, and coworker support [3]. Chou et al. [19] found caregiver satisfaction to be related to workload, team spirit, and professional support. In another study of NAs, job satisfaction was associated with job security, potential for job growth, socialization, and challenging work [20]. Thus, few similarities in results are evident. It is also clear from Table 1 that more than half of these prior studies have examined caregivers' job satisfaction using simple descriptive statistics.

## Methods

### Overview

The study is based on an analysis of data from two not-for-profit nursing homes located in Pittsburgh Pennsylvania. During the study assessment data were collected from all nursing home staff. The Job Description Index [10,21] was used to collect job satisfaction information, and additional questions were used to collect demographic information. The first wave of assessments of staff occurred concurrently at each of the two facilities during a two-week period in June of 2002.

We surveyed those caregivers who had regular contact with residents during the course of their work over the prior 2 weeks. This information was given to the research team from payroll records. A survey with 28 questions (including demographic questions and 14 job satisfaction questions) was placed in the mail-box of each caregiver. We asked that completed surveys be returned to a locked project mail-box that was placed in each facility. This same survey methodology was then repeated a further four times at six month intervals, giving a total of five waves of data.

This survey methodology was used because it was more cost-effective than using in-person interviews. This approach is also the most common methodology used for the collection of job satisfaction data. Each caregiver was compensated $10 for their time to complete each survey. A letter was also included along with the survey describing our study, stating that completion of the survey was voluntary, and indicating that individual responses would not be shared with the nursing home management. Temporary and pool workers were excluded from the study.

### Job satisfaction scale

Several job satisfaction instruments exist [22]. These include the Job Description Index [21,23], revised Index of Work Satisfaction [24], and the Measure of Job Satisfaction [7]. The second column of Table 1 lists job satisfaction instruments used in previous studies in long-term care settings.

In this investigation, we used the Job Description Index. This instrument was used because in the organizational science literature it is generally regarded as the most frequently used job satisfaction instrument [25]. This does not necessarily mean the Job Description Index is appropriate for use in the nursing home setting. Using an instrument designed for use in long-term care settings [e.g., [7,19]] would be a further refinement to our study. However, at the time we began our study (i.e., 2002) no such instrument was available.

The Job Description Index was also advantageous as it consists of relatively few questions, yet addresses a wide variety of domains (five), and uses a 7-point scale. The response items on this 7-point scale varied from: strongly disagree to strongly agree. For this analysis the response items were coded as follows: 1 (strongly disagree), 2 (somewhat disagee), 3 (disagree), 4 (neither agree nor disagree), 5 (somewhat agree), 6 (agree) and 7 (strongly agree). Negative valence items were not reverse coded, rather they were allowed to load negatively in the exploratory factor analysis (EFA).

We considered use of five job satisfaction domains to be important since we know little regarding nursing home staff and job satisfaction. With five domains we will likely increase our understanding of antecedents of job satisfaction. Flood and Scott [26] have shown that a narrow focus on single measures are misleading, and may lead to erroneous, or incomplete, conclusions. By including five such measures our approach might better capture the overall picture of job satisfaction of caregivers (although, as we note below ultimately only three domains were included in the multivariate analyses).

We were interested in using an instrument with multiple response categories, so that we could examine the relative degree of satisfaction and dissatisfaction of staff. Examining the degree of satisfaction and dissatisfaction is not possible with commonly used dichotomous scales (e.g., those asking yes or no questions). Dichotomous scales can determine satisfaction and dissatisfaction, but not how satisfied or dissatisfied respondents are.

The five job satisfaction domains included in the Job Description Index are satisfaction with: work, compensation, promotion opportunities, superiors, and co-workers. The original instrument had 14 items; one item ("My work is challenging") was dropped from this analysis (described below). The remaining 13 individual questions contained in each domain of the Job Description Index are shown in Table 3.

In addition, the Job Description Index was previously shown to have several desirable psychometric properties, including test-retest reliability above 75 percent, internal consistency of .81, convergent validity of .70, and stability across occupational groups [25]. This index was also recently successfully used in nursing homes [27].

### Independent variables

We examine the association of age, gender, race, marital status, tenure, and part time employment with job satisfaction. Staff in nursing homes were shown to have different retention needs according to their tenure. [15] Based on this finding we included tenure in our model. Following a prior study we used three categories of tenure, including working in the facility less than 1 year, between 1 and 5 years, and more than 5 years [15]. Full-time employment was associated with job overload in a previous study [28] and intent to leave [29]. We define part-time employment as working less than 35 hours per week. Finally, caregiver's perceptions of the quality of care was include based on the findings of a recent prior study [30], and was measured with a question asking whether the caregivers would recommend the nursing home for a relative or friend.

### Analytic approach

We examined the correlations between the variables (not reported). The correlations between the independent variables were small, and based on a threshold of .8 showed no problems of collinearity [31]. Also, values for regression tolerance statistics (not reported) showed no problems of multicollinearity.

Ceiling and basement effects were assessed for each item in the Job Description Index. This followed the work of McHorney, Ware, Lu, and Sherbourne [32] whereby we calculated the percent of responses with the lowest (floor) and highest (ceiling) scores. Since the Job Description Index has not been extensively used in the nursing home setting, we conducted exploratory factor analysis (EFA) to explore the extent to which the items in each domain appeared to represent the same underlying construct. Principal factors with varimax rotation was used and a factor loading criterion of .30 and uniqueness of < .90 were used to retain items.

The three factor scores were used as dependent variables in multivariate regression models. Factor scores, calculated by multiplying the item score by its factor loading, were used rather than simply summing the items for two reasons. First, the factor loadings implicitly reverse-code negative valence items. Thus, even though the work factor contains both positive and negative valance items, higher factor scores can be interpreted as indicating higher satisfaction. Since all items on the management factor were negative, this factor has a negative interpretation. Second, the resulting factor scores are uncorrelated with one another and thus represent distinct dimensions of job satisfaction. Factor scores are centered on zero and measured in standard deviation units.

Finally, we tested the assumption that the Likert-type questions produced interval rather than ordinal level measures of satisfaction by re-analyzing the data using the polychoric correlation matrix. This produced the same pattern of factor loadings as reported.

Individual caregivers were surveyed at six month intervals up to five times over a period of two and a half years. The advantage of this approach is that the data are likely representative of a longer period of time, rather than just a single point in time. Nevertheless, biases can occur in data consisting of repeat observations due to the potential correlation among the repeat observations. We therefore used generalized estimating equations (GEE) to avoid treating data from the same respondents as independent observations. All analyses were conducted with Stata Version 8.

## Results

Participation rates were similar at both facilities. About 75% of eligible staff completed surveys at each wave. There were a total of 717 surveys returned from a possible total of 953, however only 573 had complete data for all items, representing 251 unique individuals (average of 2.3 surveys per person). A descriptive profile of the caregivers with completed surveys for each wave of data collection at the two facilities is shown in Table 2.

The means and standard deviations for each of the questions in the Job Description Index are shown in Table 3 along with the results of the EFA. The overall mean ratings for each question ranged from 2.80 ("Salary and wage increases are given to those who do a good job") to 5.39 ("After a day's work, I really feel like I have accomplished something"). It should be noted that no ceiling or basement effects were identified for any item [33], and most mean scores were centered around the middle of the response scale. These results provide information to address our first question of what job satisfaction items are ranked highest by nursing home staff. That is, at least from the items we used, a relative picture of staff opinions of these items is shown. The third column of Table 3 has the overall rank of each item. In the EFA no reverse coding was used, however, to ease interpretation of the rankings we reverse coded the negatively worded items.

In the EFA, one item ("My work is challenging") did not load on any of the factors higher than .30 and was dropped from the subsequent analyses of factors associated with job satisfaction domains. Two items from the original promotion scale ("dead end job" and "chances for getting ahead") joined items from the co-workers scale to form a general 'work' satisfaction domain. These items use general terms that refer to broad aspects of the workplace and do not address career steps or promotion *per se*. The remaining promotion item joined the original management scale to form a negative valence management domain. This item was the only one to use the word 'promotion', which connotes interaction with management. The three factors were based on the scree test with factor 1 (pay) having an eigenvalue of 2.83, factor 2 (management) an eigenvalue of 1.81, factor 3 (work) an eigenvalue of 1.75, and a sharp drop off in eigenvalue to 0.345 for a possible fourth factor. These changes in domains from the original Job Description Index instrument likely represent our use of the instrument in the nursing home setting.

Regression coefficients from the GEE models examining job satisfaction are presented in Table 4. This information was used to examine our second question of what characteristics of caregivers are associated with job satisfaction. We found that males were less satisfied with work than females. Older workers were slightly more satisfied with pay (p < .001) but married caregivers were less satisfied with both pay (p = .020) and work (p = .040). NAs were less satisfied with pay than nurses (p = .090), however they were relatively more satisfied with the work (p < .001). Fulltime caregivers were less satisfied with pay than part-time workers (p < .001), but more satisfied with the work (p = .010). Caregivers who have been on the job for 1 to 5 years were generally less satisfied than those who have been there for either less than one year or more than 5 years. Finally, caregiver's perceptions of the quality of care (measured with the question that caregivers would recommend the nursing home for a relative or friend) was associated with all three job satisfaction domains (i.e., pay (p < .001), management (p < .001), and work (p < .001)).

## Discussion

Job satisfaction has important implications for nursing home staff, the nursing home industry, and quality of care. Nursing homes already employ more workers than steel producers and auto makers combined [12], yet we know little about factors associated with job satisfaction and dissatisfaction for these workers. In this research we find that, overall, caregivers are satisfied with the work and coworkers, but are less satisfied with promotional opportunities, superiors, and compensation (shown in Table 3).

Although not examining all of the same areas of job satisfaction, our results for many individual job satisfaction questions (see Table 3) are somewhat similar to those recently reported by Parsons and associates [3] and Will [33]. These authors also found most satisfaction with work and least satisfaction with pay. Studies with RNs and LPNs in nursing homes have also found dissatisfaction with pay [29]. It is not surprising that pay should be of concern. It is widely acknowledged that nursing home staff are underpaid relative to other areas of health care [34]. Moreover, NAs are often the working poor, many consisting of women, single-family, and/or minorities [35]. Yet, it is surprising that NAs seem to enjoy their work even more than nurses do. Given the often-reported poor quality of care in nursing homes, and difficult work undertaken by caregivers [36], one would assume that staff would have poor job satisfaction. Clearly, this is not the case. NAs would appear to enjoy working with residents and their coworkers. Other job satisfaction studies have highlighted the enjoyment staff receive from relationships with residents [3]. Others have also noted that work attitudes of stayers and leavers differ, with stayers valuing relationships with residents [35].

We speculate that if staff have higher job satisfaction ratings from forming relationships with residents, then facility management could promote such relationships. This could include permanent assignment to residents and teams (often called care pairs), but also more social activities. This may also benefit residents, for as Eaton [12] describes a "typical resident spends at least ninety-one of 112 waking hours a week doing nothing whatsoever." However, given the often chronic understaffing found in many nursing homes, encouraging caregivers to become involved in more social activities with residents will likely be difficult. It may also be difficult to make the case that this type of work is productive [12].

Also, it may be instructive to examine satisfaction with work over time. On the one hand, as more residents in nursing homes are admitted with terminal illness or severe dementia, then the time for staff to build relationships with residents and the ability of residents to form such relationships will likely decrease. If this influences work satisfaction, we may see even fewer staff willing to work in nursing homes. To the extent that the work in nursing homes was described as "emotional labour" [37], this may increase when residents are unresponsive. On the other hand, family members will likely remain to build relationships with caregivers.

Using the three job satisfaction domains (i.e., pay, management, and work), one robust finding is that we see workers with a tenure of more than one year are less likely to be satisfied with the pay. Katz [38] developed a model of employee turnover whereby he identified that workers perceptions of the work environment varied according to their tenure. He used three stages of perceptions of the work environment, socialization, innovation, and adaptation. In this study, we clearly see that satisfaction with pay declines after one year of employment.

The results from the three job satisfaction domains (see Table 4), also show that staff who perceived the quality of care to be high (agree with the statement that they would recommend the nursing home for a relative or friend) have higher job satisfaction than those who do not. Poor levels of satisfaction are linked to high rates of turnover [2,3]. The typical managerial response to high endemic rates of turnover among caregivers is to modify the conditions of employment (e.g., pay, promotion, communication). However, if satisfaction can be modified as a result of efforts to improve the quality of care, those efforts may also have the beneficial 'win-win' effect of reducing turnover.

This finding for quality is important. We could not find any other studies in the literature that have examined this relationship. This finding, if it holds up in further research, suggests that quality improvement projects may have an impact on caregiver morale. However, the opposite may also be true: unhappy staff may provide poor quality of care, thus placing residents at risk and also increasing the likelihood of turnover.

One further observation from our results using the three job satisfaction domains is that there is variability in the association between some caregiver characteristics and job satisfaction domains. For example, fulltime workers are less satisfied with pay but more satisfied with work than part time workers. Also, NAs are more satisfied with work, however they are less satisfied with pay than are nurses (although this finding did not reach statistical significance at conventional levels). By contrast, the association with tenure and perceived quality are consistent across domains. Researchers and others who focus on staff satisfaction need to be alert to important differences between subgroups of workers.

Clearly, we need to be careful in interpreting our findings. Although our sample size of caregivers was relatively large, this information only came from two facilities. This limits somewhat the generalizability of our findings.

## Conclusion

In summary, we find that overall nursing home caregivers are satisfied with the work and coworkers, but are less satisfied with promotional opportunities, superiors, and compensation. NAs appear particularly sensitive to pay, whereas other staff appear more sensitive to work. Of significance, we also find that caregivers who perceived the quality of care to be high have higher job satisfaction than those who do not.

## Competing interests

All authors were part of a team of researchers under a grant from AHRQ. This manuscript reflects the opinions of the authors and not necessarily those of the funding agency.

## Authors' contributions

Nicholas G. Castle, wrote the manuscript and participated in the analyses.

Howard Degenholtz, was responsible for conducting most of the analyses. He also provided text for the methods section.

Jules Rosen, edited the manuscript.

## Pre-publication history

The pre-publication history for this paper can be accessed here:



## Figures and Tables

**Table 1:** Studies of job satisfaction in long-term care settings

**Author(s)**	**Job Satisfaction Instrument**	**Number of Items**	**Number of Response options (Anchors used)**	**Job Satisfaction Domains**	**Sample Size and Setting**	**Analyses Used**	**Significant Findings**
Parsons and associates (2003) [3]	Modified from Herzberg (1966)	35	5 (strongly disagree – strongly agree)	Personal opportunity Supervision	550 NAs in 70 facilities in Louisiana	Ordinary Least Squares Regression	Most dissatisfied with pay, benefits, and recognition
				Benefits			
				Coworker support			
				Social rewards			
				Task rewards			
Moyle and associates (2003) [39]	N/A	N/A	N/A	Workplace flexibility	27 RNs and NAs in one facility in Australia	Content analysis of focus group data	Satisfaction was linked to workplace flexibility, residents, team environment, and better resident care
				Team environment			
				Optimal resident care			
Chou, Boldy, & Lee (2002a, b) [7, 19]	Measure of Job Satisfaction (MJS)	22	5 (very dissatisfied – very satisfied)	Professional support	Seventy facilities with 610 nursing home staff and 373 hostel care staff in Australia	Structural Equation Modeling	Job satisfaction is associated with professional support
				Personal satisfaction Workload			
				Training			
				Team spirit/co-workers			
Will and Simmons (1999) [33]	Job Descriptive Index (JDI)	NG	NG	Work on present job	423 NAs in 29 nursing homes in Ohio	Means	Satisfied most with work and least with pay
				Pay			
				Opportunities for promotion			
				Supervision			
				Co-workers			
				Job in general			
Atchison (1998) [20]	Job Diagnostic Survey	14	5 (extremely dissatisfied – extremely satisfied)	Satisfaction	283 NAs in 24 nursing homes	Chi square	Job satisfaction lowest for security, growth/development, socialization, and challenges
				Job security			
				Coworkers			
				Sense of accomplishment			
				Helping other people			
				Dissatisfaction			
				Pay/benefits			
				Potential for job growth			
				Management			
				Autonomy			
Kiyak, Namazi, & Kahana (1997) [27]	Job Descriptive Index (JDI)	NG	NG	Work on present job	308 nursing home and community agency staff	Ordinary Least Squares Regression	Higher dissatisfaction associated with turnover
				Pay			
				Opportunities for promotion			
				Supervision			
				Co-workers			
				Job in general			
Gillies, Foreman, & Pettengill (1996) [22]	Index of Work Satisfaction (IWS)	44	7 (not given)	Autonomy	44 nurse directors and nurse educators working in long-term care facilities	Repeated Measures ANOVA	Job satisfaction highest for interactions, autonomy, and professional status
				Interaction			
				Agency policies			
				Pay			
				Professional status			
				Task requirement			
Grieshaber, Parker, & Deering (1995) [1]				Work environment	Two nursing homes		
				Job content			
Irvine & Evans (1995)+ [40]	N/A	N/A	N/A	Routinization	Meta-analyses with combined sample size of 5,352	Meta-analyses	Work content and work environment are more strongly associated with job satisfaction than economic variables
				Autonomy			
				Feedback			
				Role conflict			
				Role ambiguity			
				Work overload			
Coward and associates (1995) [29]	Modified Stamps and Piedmonte (1986) scale [IWS]	18	5 (strongly disagree – strongly agree)	Professional status	281 RNs and LPNs from 26 nursing homes	Multivariate regression analysis	Five factors associated with job satisfaction (race, income, supervisor, initial intent to stay, current intent to leave)
				Task requirement			
				Autonomy			
				Interactions with other nurses			
				Pay			
Monahan & Carthy (1992) [41]	N/A	N/A	N/A	Attachment	75 NAs at 7 nursing homes	Content analysis	Attachment most related to retention of NAs
				Gratification			
				Demands			
				Monetary needs			
				Decision-making			
Grau and associates (1991) [42]	Combined several scales	44	5 different scales	Job process	219 NAs in one nursing home	Hierarchical regression analysis	Social atmosphere and job benefits associated with institutional loyalty
				Attitudes toward administration			
				Social atmosphere			
				Job benefits			
				Job tasks			
Anderson, Aird, & Haslam (1991) [43]	NG	12	5 (strongly disagree – strongly agree)	None	212 nursing staff in 6 nursing homes	Means	Nursing staff have high levels of satisfaction, but is associated with absenteeism
Humphris & Turner (1989) [44]	Porter (1962) scale	13	6 (extremely satisfied – extremely unsatisfied)	Working conditions	84 nurses at a unit for the elderly severely mentally infirm	Chi square	Low satisfaction was associated with turnover from unit
				Emotional climate			
				General			
Mullins and associates (1988) [45]	Job Satisfaction Survey (JSS)	36	NG	Pay	Heads of departments (n = 439) from 46 nursing homes	Regression analyses	Most satisfied when individual efforts are rewarded
				Promotion			
				Supervision			
				Benefits			
				Rewards/appreciation			
				Working conditions			
				Coworkers			
				Nature of job			
				Communication			
Deckard, Hicks & Rountree (1986) [46]	Job Diagnostic Survey (JDS)	NG	NG	Skill variety	340 nurses from a nursing home chain	Means	Job satisfaction was similar to norms in other occupations
				Task identity			
				Task significance			
				Autonomy			
				Job feedback			
Waxman and associates (1984) [47]	Minnesota Satisfaction Scale	20	5 (very dissatisfied – very satisfied)	Job Satisfaction Scale	234 NAs in 7 facilities, uses 20 questions for overall job satisfaction score	Kendal's Rank Order Correlation	Positive association between job satisfaction and turnover
Bergman et al. (1984) [2]	None	19	4 (none – very much)	Job	12 long-term care facilities and 432 RNs, LPNs, and NAs	ANOVA	Descriptive results provided
				Knowledge, skill, and attitudes			
				Autonomy			
				Stress			

NA = Nurse Aide; RN = Registered Nurse; LPN = Licensed Practical Nurse

+ = This study is a meta-analysis, and does not include only long-term care studies

NG = Not given; N/A = Not applicable

**Table 2:** Staff characteristic descriptive statistics

	Wave 1	Wave 2	Wave 3	Wave 4	Wave 5	Unique Individuals
	(n = 124)	(n = 106)	(n = 115)	(n = 113)	(n = 115)	n = 251
	
Age (mean; [sd])	41.6 [10.7]	40.9 [11.2]	42.2 [11.3]	41.5 [10.9]	40.6 [11.9]	38.8 [11.8]
	
Gender (% Male)	8.1	5.7	8.7	7.1	7.0	10.4
Race (% African American)	39.5	41.5	44.4	44.3	47.0	47.0
Marital status (% Married)	38.7	41.5	44.4	43.4	40	37.1
Job category (% Nurse Aides)	59.7	64.2	60.0	59.3	61.7	61.4
Fulltime (%)	85.5	84.0	82.6	71.7	68.7	75.7
Tenure:						
Less then 1 year	33.1	34.0	26.1	31.0	36.5	52.2
1 to 5 years	29.8	37.7	43.5	42.5	37.4	24.3
Over 5 years	37.1	28.3	30.4	26.6	26.1	23.5
Perceived High Quality	54.0	43.4	57.4	52.2	60.0	55.8

**Table 3:** Item means and factor loadings for job satisfaction

			**Factor Loadings**		
			
**Items (Original JDI domain)**	**Item Mean (SD)^^^**	**Rank**^+^	**Pay (Positive)**	**Management (Negative)**	**Work (Positive)**
Positively Worded Items:					
After a day's work, I really feel like I have accomplished something (w)	5.39 (1.58)	1			0.54
Working for this Facility is like being part of a family (w)	4.13 (1.96)	6			0.58
I am paid fairly for the work I do (c)	3.31 (2.01)	11	0.70		
Salary and wage increases are given to those who do a good job (c)	2.80 (1.91)	13	0.52		
My pay is better than that for similar jobs in other nursing homes (c)	3.15 (1.83)	12	0.63		
My chances for getting ahead in this facility are good (p)	3.85 (1.90)	7			0.44
The people I work with are stimulating (cw)	4.25 (1.63)	5			0.48
Negatively Worded Items:					
I just hate to get up in the morning to go to work (w)	3.49 (1.90)	3			-0.31
I am in a "dead end" job (p)	3.60 (1.98)	4			-0.40
My opportunities for getting promoted in this facility are somewhat limited (p)	4.50 (1.87)	9		0.33	
The people I work with are unpleasant (cw)	3.30 (1.90)	2			-0.39
Management is quick to criticize poor performance (m)	4.63 (2.01)	10		0.65	
Management is hard to please (m)	4.35 (1.89)	8		0.67	

Note: Principal components with varimax rotation. Original domains: w = work; c = compensation; p = promotion; cw = co-workers; m = management. Eigenvalues for Pay = 2.83, Management = 1.81, and Work = 1.75; Cronbach's Alpha for comparable unit-weighted scales: Pay = .60; Management = .71; Work = .70; JDI = Job Descriptive Index; n = 574; ^^ ^= Response items were coded 1 (strongly disagree), 2 (somewhat disagee), 3 (disagree), 4 (neither agree nor disagree), 5 (somewhat agree), 6 (agree) and 7 (strongly agree); ^+ ^No reverse coding was used for the EFA, however, the rank was computed by reverse coding negatively worded items and listing from highest score (listed as number 1) to lowest score (listed as number 13).

**Table 4:** Staff characteristics associated with job satisfaction

	**Pay (+)**			**Management (-)**			**Work (+)**		
			
	Coef.	Std. Err.	P > |Z|	Coef.	Std. Err.	P > |Z|	Coef.	Std. Err.	P > |Z|
**Age**	0.010	<0.001	<0.001	-0.010	<0.001	0.060	<0.001	<0.001	0.360
**Gender (Male)**	-0.030	0.120	0.830	-0.040	0.120	0.720	-0.250	0.110	0.030
**Race (African American)**	0.070	0.060	0.300	0.010	0.070	0.840	0.190	0.060	<0.001
**Marital status (Married)**	-0.160	0.070	0.020	0.010	0.070	0.840	-0.140	0.060	0.040
**Job category (Nurse Aide)**	-0.110	0.070	0.090	0.030	0.070	0.710	0.250	0.060	<0.001
**Employment (Full-time)**	-0.270	0.080	<0.001	0.040	0.080	0.580	0.190	0.070	0.010
**Tenure **^**a**^									
**1 to 5 years**	-0.320	0.080	<0.001	0.200	0.080	0.010	-0.140	0.070	0.050
**> 5 years**	-0.240	0.090	<0.001	0.290	0.090	<0.001	-0.120	0.080	0.150
**Perceived High Quality**	0.420	0.060	<0.001	-0.210	0.070	<0.001	0.590	0.060	<0.001

Note: n = 574; Reference categories are: female, white, not married, nurse (Registered Nurse or Licensed Practical Nurse), part-time, less than 1 year, and perceived low quality.
